# Epidemiology of Alzheimer's Disease and Dementia in Arab Countries: A Systematic Review

**DOI:** 10.1155/2019/3935943

**Published:** 2019-10-29

**Authors:** Ashraf El-Metwally, Paivi Toivola, Mashael Al-Rashidi, Shanila Nooruddin, Munazza Jawed, Raghad AlKanhal, Hira Abdul Razzak, Nada Albawardi

**Affiliations:** ^1^College of Public Health and Health Informatics, King Saud Bin Abdulaziz University for Health Sciences, Riyadh, Saudi Arabia; ^2^King Abdullah Specialist Children Hospital, King Abdulaziz Medical City, Riyadh, Saudi Arabia; ^3^Saudi Food and Drug Authority, Riyadh, Saudi Arabia; ^4^Dow University of Health Sciences, Karachi, Pakistan; ^5^King Abdullah International Medical Research Center/King Saud bin Abdulaziz University for Health Sciences, Ministry of National Guard-Health Affairs, Riyadh, Saudi Arabia; ^6^Ministry of Health and Prevention, Dubai, UAE; ^7^Epidemiology and Biostatistics Section, Health Sciences Research Centre, Princess Nourah bint Abdulrahman University, Riyadh, Saudi Arabia

## Abstract

**Background/Objectives:**

Contrary to popular belief, the condition of dementia is not an actual discrete disease, but rather a group of symptoms, most notable of which is the disturbance of memory and social ability, often severe enough to impair daily functioning. As a result, it has been a major cause of functional deterioration among varying populations in the world. This study is aimed at reviewing the epidemiology of dementia in Arab countries in terms of its prevalence, distribution, and risk factors.

**Methods:**

A systematic literature review was conducted using articles published in PubMed, Embase, Scopus, and other local journals between 1990 and 2018. After applying the inclusion and exclusion criteria, a total of 18 studies were concluded to be eligible for the review.

**Results:**

Prevalence studies demonstrated that dementia is indeed a prevalent condition in Arab countries, ranging between 1.1% and 2.3% among age groups of 50 years and older, as well as between 13.5% and 18.5% among age groups of 80 years and above. However, these results are not different from those of many other countries in the world. Moreover, prevalence was found to vary depending on sociodemographic characteristics. Major risk factors of dementia included hypertension, low income, and low education, while the risk of developing dementia is increased by obesity, diabetes mellitus, and cardiovascular risk factors. Despite the growing evidence regarding the epidemiological distribution and determinants of dementia worldwide, studies from the Arab region remain scarce.

**Conclusion:**

This systematic review highlights the need for population-based studies to provide necessary information for developing preventive and curative strategies specific to the Arab region.

## 1. Introduction

Alzheimer's disease (AD) and its related disorders are conditions characterized by a gradual and unpreventable deterioration of multiple cognitive functions (including complex attention, executive function, learning and memory, language, perceptual-motor, or social cognition from a previous level of performance), serious enough to interfere with the daily lives of those affected in both social and professional terms [1, 2]. One of the special characteristics of AD is dementia, a “neurocognitive disorder” or NCD, according to DSM-5. These disorders indubitably have a major negative impact on the lives of the patients, their families, and society [3, 4]. The World Health Organization (WHO) determined that dementia can currently be found among 36 million of the world's population with a progressive increase in occurrence over the past decades. The WHO also estimates that this number will be doubled within the next 20 years [5]. Similarly, the global number of AD patients was 44 million in 2015; however, this number is expected to triple, reaching 115 million individuals by 2050 [6]. Every year, around 4.6 million new dementia cases are added in the existing pool, the highest growth projections being found in China along with its South Asian neighbors [7]. Nevertheless, previous evidence indicates that dietary and lifestyle modification could reduce the risk of dementia and its comorbidities such as type 2 diabetes, hypertension, obesity, and psychiatric behavioral changes [8, 9].

Epidemiological research studies have constantly shown differences in the rates of dementia among different geographical and ethnic populations and other sociodemographic characteristics. Compared to countries in Europe and North America, lower dementia rates are reported in countries such as those in Africa and Asia, specifically Singapore, Japan, China, Nigeria, and India [10]. In the United States, the occurrence of AD was found to be 1.6% among the 65-74-year age group, followed by 19% in the 75-84-year age groups and up to 42% among those who are 85 years old and above [11]. Thus, it can be easily concluded that one of the most significant risk factors for dementia is age [1]. The WHO also reported that the average aging population's (85 years and more) growth rate will increase by 5% between 2000 and 2050 in some Arab countries [12]. Therefore, age differences between populations often result in varying estimates of the prevalence, making it difficult to accurately compare them. Furthermore, vascular risk factors and genetic factors (apolipoprotein E) also play a significant role. Indeed, the prevalence of dementia among the elderly is significantly higher than that among individuals aged 20 to 50 [1]. A meta-analysis showed strong evidence for the association of dementia with statin, estrogen, nonsteroidal anti-inflammatory drugs (NSAIDS), and antihypertensive medications, while folate, coffee, and vitamins C and E were found to be protective for AD [13]. Moreover, depression, hypertension, atherosclerosis, arthritis, and diabetes significantly increase the risk of AD. Likewise, lifestyle factors including high BMI and low levels of education were among other risk factors identified. On the other hand, current smoking, cognitive activity, and light-to-moderate drinking decrease this risk [13].

Epidemiological studies have made it possible to highlight the complexity of these disorders and to identify major characteristics that have a direct influence on their distribution. Like other countries in the world, the Middle East has undergone many considerable changes that have had a direct influence on the population's health issues. One major change highlighted by Simpson [2] is that noncommunicable diseases replaced infectious diseases as the main health concern within the region. Findings reveal that 47% of the total burden of disease is attributed to noncommunicable diseases, with a steep rise to 60% expected by 2020 [3]. A limited amount of data is currently available from Middle Eastern countries about Alzheimer's and dementia. Moreover, no clear evidence for the occurrence of AD following the increase in the aging population has been generated.

A dementia report from the WHO has revealed an alarming increase in dementia across the Middle Eastern region, where prevalence may have an increase of 125% by 2050 [14]. Several studies have been conducted in Arab countries to obtain data on the prevalence, risk factors, prevention, and management of AD and dementia, but no review had yet been conducted to systematically summarize current existing evidence. Therefore, we aimed to fill this gap in the literature by carrying out a systematic review to evaluate the epidemiology of dementia in Arab countries, with a clear focus on its prevalence, distribution, and risk factors.

## 2. Methods

### 2.1. Data Collection Strategy

Studies from 1990 to 2018 were systematically retrieved and extracted from PubMed, Scopus, and Embase. Detailed searches in local journals and cross-referencing were also performed to enrich the investigation and to broaden the scope of the research. A combination of Medical Subject Heading terms and search terms were utilized, including “AND” and “OR” Boolean operators to retrieve the targeted results. The key terms used in the search include “Epidemiology”; “Prevalence”; “Complications”; “Risks”; “Dementia”; “Alzheimer's Disease (AD)”; “Arab Countries”; “Arab World”; “Incidence”; “Prognosis”; “Saudi”; “Kuwait”; “Egypt”; “Syria”; “Lebanon”; “Libya”; “Sudan”; “Algeria”; “Jordon”; “Tunisia”; “Iraq”; “Morocco”; “Qatar”; and “Bahrain.”

### 2.2. Study Eligibility Criteria

An inclusion criterion was applied to the article title and abstract. Articles providing information on the prevalence or risk factors of dementia and Alzheimer's disease in one or more selected Arab regions were included. Additionally, primary research articles published in English in peer-reviewed journals between the late 1990s and 2018 were extracted to be included in the study. On the other hand, studies including nonrelevant regions and populations were excluded.

### 2.3. Identification and Study Selection

Research studies were selected and reviewed using a three-step process. Firstly, two of the researchers independently selected the studies that matched the standards for selection by browsing through the retrieved abstracts and titles. Secondly, the two researchers proceeded to discretely read potentially eligible studies. The outcome of these steps of the review demonstrated no disagreements or inconsistencies within the selection of studies. Finally, the lists of references of the studies included were scanned to identify additionally relevant research papers, completing the third step of the process. The selection of articles followed the Preferred Reporting Items for Systematic Reviews and Meta-Analyses (PRISMA) guidelines.

### 2.4. Data Analysis

**Figure 1:**
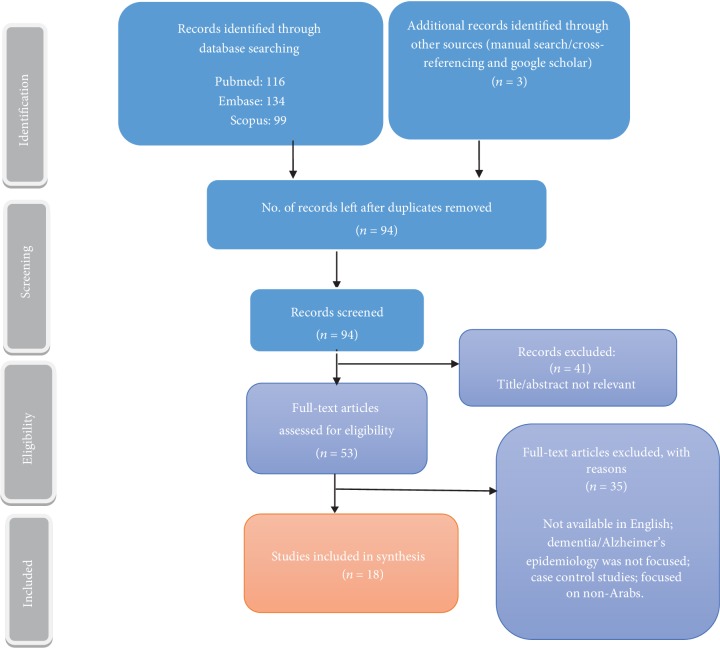
Retrieval of article and screening process.

A total of 352 articles (116 from PubMed, 134 from Embase, 99 from Scopus, and 3 through cross-referencing/local journals) were identified in the original search. Articles that formed duplicates were then removed, and the remaining papers were further scanned based on their relevance to the topic. To apply the inclusion criteria, titles and abstracts of each article were reviewed thoroughly. All articles with insufficient information, unrelated topics, or nonrelevant populations were excluded. A total of 53 full-text articles were assessed for eligibility. Finally, we were able to select 18 pertinent articles that conformed to the inclusion criteria and provided succinct information which met the aims and objectives of the study. Afterwards, the data collected was summarized through narrative with an overview of the year, country, outcome measure, setting/sampling technique, study design, sample size, and study key findings. This was then followed by a synthesis of the selected studies based on the outcome measures.  Figure 1 represents the process of the research.

### 2.5. Risk of Bias Assessment

We adopted the quality assessment score published by Prince et al. [15]. It includes four quality assessment norms with a focus on the key areas leading to potential bias in observational studies, including sample size, study design, response proportion, and diagnostic assessment. The overall quality was assessed by summing the scores up. Sample size estimation is undoubtedly an important factor for the study design; it should be sufficient for a proper estimation of the prevalence for a geographic area in published studies. Similarly, the study population should also be correctly classified based on the conditions present with a specified confidence interval and sampling error [16].

## 3. Results and Discussion

### 3.1. Overview of the Included Studies

The PRISMA flowchart (Figure 1) presents the results of the screening and selection process of the articles identified. The systematic literature search retrieved 352 studies, of which 18 were eventually found to meet the objectives of the review. These studies were sorted into different categories depending on prevalence, frequency of sub-types, and associated risk factors. Thirteen of the studies reported on the prevalence and frequency of dementia in the Middle East, four of which were hospital-based studies. Furthermore, eleven of the publications discussed the risk factors associated with dementia (Tables 1, 2, and 3).

### 3.2. Quality Assessment of the Included Studies

Out of the eighteen studies which were included in this systematic review, only five studies had a sample size of more than 1,500, while almost half of the studies had a sample size of less than 500. Most of the studies were conducted in multiple stages, but they had no samplings of screen negatives or weighing back. Only 50% of the studies applied proper sampling techniques. Studies by El Tallawy et al. [18] involved door-to-door screenings of individuals with a maximum sample size of the general population. About 30% of the studies did not mention the response rate, while others extracted data from the hospital records retrospectively. Regarding the diagnostic assessment, around 45% of the studies involved multiple strategies for better and more definite diagnoses of dementia and Alzheimer's disease. For the reviewed studies, we gave the quality score ranging from 0 to 11. The studies were categorized as low quality (score 0-4), medium quality [5–8], and high quality with scores [9–11]. Overall, the studies from Egypt, Saudi Arabia, and UAE showed the highest scores (Table 4).

### 3.3. Prevalence

Studies about the prevalence and frequency of dementia and its subtypes were conducted across six countries; four studies in Egypt [17–20], two in Lebanon [21, 22], three in Saudi Arabia [3, 26, 27], two in Qatar [23, 28], one in Tunis [24], and one in the United Arab Emirates (UAE) [25]. These studies were published between 1990 and 2018 and included sample sizes ranging from 77 to 62,583 subjects. The settings for the studies included households, nursing homes, and healthcare facilities. The methodologies used were heterogeneous from the use of cross-sectional studies with a single phase of screening or diagnostic testing using only interviews or questionnaires to multistage testing comprising clinical examination, laboratory, and radiological investigations. Other studies reviewed hospital records of both in-patient and out-patient departments and described hospital frequencies of dementia and its subtypes. Study populations also varied widely among different research articles, with some including all members of households and others limiting evaluations to those over 50 or 60 years of age.

### 3.4. Risk Factors

#### 3.4.1. Age

The association between age and dementia was reported in five studies. A secondary analysis was conducted using random sample data of elders from two Lebanese governorates in a cross-sectional study [22]. The study reported significantly increased odds of dementia with increased age [OR: 75–84 years = 4.00 (95% CI: 1.46, 10.95); OR 85+ years = 7.07 (1.84, 27.03)] [29]. Similarly, two other cross-sectional studies revealed age-specific dementia prevalence, which was projected to double every 5 years in a sample from Egypt [17]. On the other hand, Ouanes et al. [24] further noted no significant association between age and the prevalence of dementia among nursing home residents in Tunisia.

#### 3.4.2. Education

Education was reported to be significantly associated with dementia in four studies. El Tallawy et al. [19], who conducted a cross-sectional study among ≥50-year-old Egyptians, reported a significantly higher prevalence of dementia among illiterate participants compared to educated ones (3.6% vs. 79% respectively, *p* < 0.001). In another cross-sectional study, Khedr et al. [20] reported a greater prevalence of dementia in illiterate elderly participants as opposed to their literate counterparts (10.12% vs. 2.25%; *p* = 0.00001) within a random sample of households in Egypt.

In one particular case, which was a control study using subjects from senior homes and a university hospital in Jordan, Al-Khateeb et al. [30] found a 6.29-fold (*p* = 0.002) rise in the risk of developing dementia for illiterate subjects. In another cross-sectional study, Chaaya et al. [29] reported that the odds of dementia were significantly higher (OR: 3.39, 95% CI: 1.71, 6.70) among those with no formal education in Lebanon (formal education including those who went to school, technical institute, or university vs. no formal education or no enrollment in any educational system including those who read and write or were illiterate). Only one cross-sectional study reported that education level was not significantly associated with dementia [24].

#### 3.4.3. Gender

Two cross-sectional studies reported on the association of dementia and gender. Chaaya et al. [29] found that being female increased the odds of developing dementia by no less than 2 times. However, when controlling for all other factors (such as, age, marital status, education, and income), the outcomes were not statistically significant. Likewise, Ouanes et al. [24] found that gender did not differ significantly between nursing home residents with or without dementia.

#### 3.4.4. Health Conditions

Chaaya et al. [29] assessed the association between dementia and cardiovascular disease (CVD) risk factors and found that uncontrolled hypertension (vs. no hypertension) was the sole significant risk factor that exacerbated the odds of developing dementia [OR 6.35 (95% CI: 1.60, 25.10)] among elderly participants. Among elderly Saudi subjects from an outpatient clinic, Alaama et al. [31] reported in a cross-sectional study that diabetics were significantly more likely to have cognitive impairment than those who were not diabetic (16% vs. 3%, *p* < 0.005). Moreover, no statistically significant difference was found among diabetic patients for glycemic control or the duration of diabetes on cognitive test results. In a case-control study, Al-Khateeb et al. [30] also demonstrated no significant correlation between serum copper, lipid profile, and cognitive decline among elderly Jordanians.

#### 3.4.5. Genetic Factors

Haithem et al. [32] conducted a case control study in Tunisia to examine the association between five genetic risk factors for dementia, studied separately or assembled in haplotypes. All the polymorphisms studied, excluding PON1-Q192R, were found to be significant. Shamieh et al. [34] explored the distribution of APOE genotypes within the general Lebanese population in a cross-sectional study. They found that the prevalence of these genotypes in Lebanon was similar to that in Asian populations. There was a significant difference in APOE genotypes in AD patients, controls, and the general Lebanese population, where the E4 allele was 3 times higher in AD patients.

#### 3.4.6. Other Factors

Other variables associated with cognitive decline or risk for dementia included insufficient personal income (OR: 3.90, 95% CI: 1.58, 9.60) [29], while in another cross-sectional study, occupation and residence had no significant effect on the prevalence of dementia [17]. Regular consumption of coffee (drinking coffee nearly every day) showed a protective effect of a 6.25-fold decrease in the risk of cognitive decline [30]. In Egypt, Khedr et al. [20] reported that the CPRs were considered to be more significant in urban than in rural areas (7.1 versus 3.27/100, respectively; *p* = 0.03) and in industrial areas than in nonindustrial areas (13.23 versus 1.99; *p* ≤ 0.001).

### 3.5. Discussion

In the current systematic review, we aimed to evaluate the prevalence, distribution, and risk factors of dementia and AD in Arab countries. A total of 18 studies were included in this review. Among them, five were classified as high quality, six as medium quality, and seven as low-quality studies according to our assessment criteria.

Dementia and its subtypes were found to be prevalent in Arab countries as shown by our systematic review. However, there is a lack in population-based studies in the Arab world. There was a lower number of studies that examined the prevalence and risk factor of dementia and AD in most of the Arab countries, with particularly limited estimates for recent years. One of the most common risk factors associated with it was age. High occurrence of cognitive decline, AD, and dementia were found to be significantly associated with increasing age. Besides age, another notable risk factor was education, showing higher odds of dementia in illiterate compared to educated people. Moreover, females were at a higher risk of developing dementia, AD, and cognitive decline than males. Health conditions of the patients like hypertension, cardiovascular diseases, diabetes, and depression were also among the identified risk factors for dementia in Arab countries. This review also picked a few other factors which were found to lower the odds of developing dementia, namely, regular utilization of coffee and smoking.

The reviewed literature on the prevalence of dementia and AD in various Arab countries shows that Egyptian studies reported a prevalence of dementia that was around 2-2.26% [17–20], 3.34-7.4% in Lebanon [21, 22], 5.2-3.85% in the KSA [3, 26, 27], 1.1% in Qatar [28], 58.4% in Tunisia [24], and 3.6% in the UAE [25]. The literature includes various other studies that demonstrated the prevalence of AD and dementia in their respective populations. The estimates of prevalence of vascular dementia in Latin America are 0.9% for a study in Brazil [35], 2.1% in Venezuela [36], and 1.9% in Cuba [37], while similar frequencies were observed between 0.6% in Sri Lanka and 2.1% [38] in South Korea [39]. In a study from Istanbul, AD prevalence was 11%, specifically among people aged 70 years and above [40], whereas more recent evidence shows a 6.4% AD prevalence, as reported in a study conducted on the Turkish population [41]. Similarly, a study conducted on the Arab population of Israel showed a higher prevalence rate of vascular dementia 5.9% [42]. Ertekin et al. [41] further point out that AD is more common in elderly. This is fully justified because individuals belonging to the 75-84-year age group had ten times the AD prevalence of those aged 65-74 years. Moreover, the risk of developing AD for people aged 85 years and above was 36 times higher than that of the 65-74 age group, with an overall AD risk to be increased 17-fold with advancing age. The major driver of this projected increase is population aging. On a similar note, a meta-analysis including 17 studies concluded that the occurrence of dementia was 2.2-8.4% at 65 years old and over, 10.5-16% at 75 years old and above, and 15.2-38.9% at 85 years old and over [37]. Other drivers for this projected increase may include lack of knowledge about dementia, low literacy levels, and culture. Elderly adults are highly respected within Arab culture, and placing one's elderly relatives somewhere outside their home is considered an abandonment of family duty. Often, the dementia carries stigma with it. Therefore, it is widely believed that dementia is caused by fate.

AD is more common in women than in men [41]. Incidence studies for dementia report no difference of risks between males and females [43]. However, the prevalence studies have shown females to be more vulnerable to developing dementia [1, 44]. Another large population-based study proposed no specific gender differences in the dementia incidence, yet after 90 years of age, the AD occurrence was higher in women, while the vascular dementia proportion was found to be higher in males than in females across all age groups [45]. Additionally, having a lower level of education was also recognized as one of the most established risks for dementia and AD [46]; this risk being similar in males and females [47].

Bowirrat et al. also report associated risk factors of dementia among the Arabs of Israel, which were being female, older age, and illiteracy [48]. Effects of education were assessed in very heterogeneous ways across studies (such as formal or no formal education; illiterate vs educated; <12 vs >12 years of education), and therefore, we could not compare their results. But as most researchers used years of education, we can say that dementia and years of education are inversely related to each other. Similar relation was also found in different population studies like Dong et al. [49], Ott Alewijin et al. [50], and Kalaria et al. [51]. These finding are consistent within Arabs living in non-Arab countries [52]. Drinking 3 to 5 cups of coffee per day was associated with a 65% reduced risk of AD/dementia at a later stage [53]. The Rotterdam study suggested that smokers have a greater risk of dementia and AD [54]. Depression was also proposed to be a risk factor and a prodrome of dementia [55].

This review also demonstrated the enhanced risk of developing AD in preexisting chronic diseases. Likewise, an increase in the rate of AD was noted by Ertekin et al. [41]. The authors revealed a 4.1-fold increase among diabetics, a 3.9-fold one among those with Parkinson's disease, and a 2.6-fold one in cardiac patients, as well as a 4.2-fold increase in those people who had a history of dementia in their families. Saedi et al. [56] supplied strong evidence on the association of diabetes with increased risks of dementia and cognitive impairment. Biessels et al. [57] and Kravitz et al. [58] provided considerable insights on dementia as the most chronic complication of diabetes mellitus, occurring commonly at an older age [59]. The association between vascular dementia and diabetes was found to be 100-160% as compared to AD (45%-90%) [60], further advancing the danger of cerebrovascular accidents [61]. Ojo and Brooke [62] also evaluated the shared pathogenesis of AD and diabetes and argued about the compromised overall quality of life through weakening self-management that continues in a vicious cycle.

The study also reported a significant association of cardiovascular risk factors and the prevalence of dementia. The risk and incidence of dementia increase when a person is prone to smoking, alcohol, and obesity. These results are comparable with the studies conducted by Deckers et al. [63] and Suemoto [33]. Bowirrat et al. [48, 64] stated that higher prevalence rates of dementia in a certain population can be due to genetic factors, which undeniably form the major risk factor for the incidence of the disease in that given community. Similarly, another study on the Arab population living in Wadi Ara reported higher odds of Alzheimer's disease associated with hypertension (OR = 2.08; 95% CI: 1.18–3.65) [65]. Suemoto [33] argued that with aging, the risk of diagnosing vascular dementia among the elderly is reduced, but the risk of developing AD increases. Therefore, the vascular factor is especially dangerous across Arab countries, in which a significant part of the population simply does not survive the onset of dementia of a different type [66]. According to Rizzi et al. [67], mixed dementia results when cerebrovascular pathologies combine with AD. This implies that each pathology has its own effect on the continuum for dementia, whereby the pure AD and pure cerebrovascular disease form the two extremities of it.

#### 3.5.1. Strengths and Limitations

Although adequate efforts have been made to include, retrieve, and search for all relevant articles, we were still unable to absolutely claim to have unearthed all potentially relevant articles for inclusion in this review. The exclusion of studies that were available in languages other than English also served as a limitation. Publication bias, which refers to the tendency to publish positive findings of the manuscripts, is a potential limitation of our study. The different factors responsible for causing variations in the results of selected studies such as genetic factors, methodological issues, socioeconomic characteristics, and the mortality rates among elderly population are also considered limitations. The more variation found between the studies in terms of population setting, etc., the more difficult it would be to compare these studies and combine their results for analysis. Traditionally, many studies explored the occurrence and epidemiology of dementia, but this review is the first of its kind to combine the findings systematically among different Arab countries.

## 4. Conclusion

This paper provided an overview of the burden of dementia in Arab countries. There have been only a limited number of primary studies conducted in the Arab countries. The evidence gathered from the previous literature suggested that patterns of dementia in the Arab countries are not different from the rest of the countries. However, the prevalence varies from lower sociodemographic status (SES) towards higher SES. The major risk factor that exacerbates dementia among the Arab countries is age and illiteracy. Genetic factors, diabetes mellitus, and CVDs have been identified to also be associated with dementia. Gender differences have not been reported, but a few prevalence studies indicated that females were at a higher risk of dementia, whereas vascular dementia as more common among men. Higher prevalence of dementia and its common risk factors were observed not only among Arabs residing within Arab countries but also among Arab populations living in other countries as well. It is clear from the review that the epidemiology of dementia forms a critical issue that offers a higher scope for research. Future studies should focus on the epidemiology of dementia and other risk factors associated with it, taking into consideration the diversity specific to this region, since the respective differences should be considered in planning healthcare services. The risk factors often vary depending on cultural traditions, social features, demographics, and economic privileges. These differences make the Middle East an important area of research for conducting epidemiological studies to determine the prevalence of certain diseases.

### 4.1. Implications of Findings for Future Research

A major drawback identified within this study is that dementia is not thought of as a major healthcare challenge [68]. Rather, it is considered a normal aging process [44]. The current demographic transitions of dementia in Egypt have rendered it a future challenge [69]. Similarly, according to the Alzheimer's Disease International (ADI) Delphi consensus study, it was estimated that 71% of the people living in developing countries will be suffering from dementia by 2040, which is alarming [70]. The Middle East is characteristically different from other regions in the world in terms of its diversity and heterogeneity in religion. In the Middle East, the elderly population lives among family, with their children and newer generations Therefore, family members should be taught about the symptoms of dementia and how to care for the elderly affected by it. Neuropsychiatric and psychogeriatric specialties can and should play a lead role in this. However, general healthcare should also include the care for AD. The knowledge obtained from this review, and other similar reviews from Arab countries would help public healthcare practitioners and policy makers be able to understand the occurrence and major exacerbating factors related to dementia so as to set priorities and design plans for specialty care for patients with dementia and AD. Longitudinal studies will further aid in understanding the course of diseases, additional symptoms, and problems that can occur with reference to dementia and AD.

## Figures and Tables

**Table 1:** Studies of dementia prevalence.

Author	Year	Country	Study design	Criteria used for dementia identification	Cut-offs used for dementia diagnosis	Sample size	Setting/sampling tech	Study key findings
Farrag et al. [17]	1998	Egypt (Assiut governorate)	3-phase cross-sectionalpopulation-basedstudy	Modified MMSE Clinical diagnosis Lab investigations	Positive MMSE ≤ 21 Subjects scored 21 or less were eligible to proceed into phase 2 for clinical diagnosis Negative MMSE > 21 Scores 21 to 17 for mild; 16-9 for moderate; less than 9 for severe degrees of dementia	2000	Household Systematic random of total elderly population age range > 60 years (130,000 subjects)	CPR—4.5 cases per 100 population prevalence: AD—2.2 Multi-infarct dementia—0.95 Mixed dementia—0.55 Secondary dementias—0.45 Age-specific prevalence tends to double every 5 years after age of 75 years and above The prevalence of dementia of all types for the age group: 60-64 years: 1.41 % 65-69 years: 1.87 % 70-74 years: 4.06% 75-79 years: 6.46% 80-84 years: 14.87% 85 years or over: 22.01%

El Tallawy et al. [18]	2010	Egypt (Al Kharga district)	Cross-sectional	Short standardized Arabic screening test; MMSE	MMSE score: 21–17 for mild, 16–9 for moderate, and <9 for severe degree of dementia	8,173	Household All residents (62,583 subjects) of whom 8,173 were of ≥50 years of age	Prevalence: >50 years—2.26% >80—18.48% Dementia subtypes: AD—51.2% VaD—28.7% Due to general medical conditions—12.8% Due to multiple etiologies—7.3% Degree of dementia according to MMSE scores: Mild—53.7% Moderate—38.4% Severe—7.9%

El Tallawy et al.[19]	2014	Egypt (Al-Quseir city)	Cross-sectional	MMSE Clinical exam investigations	A score of 17-21 for mild, 9-16 for moderate, and less than 9 for a severe degree of dementia	4,329	Household All residents (33,285 subjects) of whom, 4,329 were of ≥50 years of age	Total—2.01% Age: 50 ≤ 60—.27% 60 ≤ 70—1.7% 70 ≤ 80—6.5% >80—13.5% Dementia subtypes: AD—48% VaD—36% Due to general medical condition—12% Due to multiple etiologies—3%

Khedr et al. [20]	2015	Egypt	Cross-sectional	MES MMSE Neurological examination	MES total score 75–62 appear to be the range for patients with MCI A score of 17–21 on MMSE for mild, 9–16 for moderate, and less than 9 for a severe degree of dementia	691	Household Multistage random sampling	MCI—1.74 cases per 100 population Dementia—5.07 cases/100 population AD—1.74 cases/100 population VaD—1.3 cases/100 population Parkinson's—1.01 cases/100 population

Chahine et al. [21]	2006	Lebanon	Cross-sectional	MMSE and GDS	Dementia diagnosed at score < 25 25–29—nondemented but with indeterminate cognitive function Mild, moderate, or severe dementia—20–24, 14–19, <14 Score of 30—normal cognitive function	117	Nursing home residents (NSR) Random	Dementia of some kind found in 61 NSR (59.8%) Mild dementia in 17 NSR (27.9%) Moderate dementia in 14 NSR (22.9%) Severe dementia in 30 NSR (49.2%) Depression in 45 NSR (57.7%)

Phung et al. [22]	2017	Lebanon	Cross-sectional	RUDAS	Scores ranging from 0 to 30; higher scores indicate better cognitive function	502	Household multistage cluster sampling	Crude dementia was less prevalent (7.4%) as compared to age-standardized dementia (9%). Based on estimated total number of Lebanese older than 65 years in 2013 (404,274 persons), the estimated number of people with dementia in Lebanon was 29,916 persons

Ghuloum et al. [23]	2011	Qatar	Cross-sectional	Designed screening questionnaire Psychiatric assessment	—	1660 (297 were >50 yrs)	15 primary healthcare clinics All 18-65 years old	Prevalence: Total—1.1% (M—1.8, F—.6), above 50 years

Ouanes et al. [24]	2014	Tunis	Cross-sectional	MoCA test	Scores on the MoCA range from zero to 30, with a score of 26 and higher generally considered normal	77	Manouba nursing home All 116 residents (mean 72.6 years ± 10.3)	Dementia—45(58.4%) Only three were already diagnosed

Ghubash et al. [25]	2004	United Arab Emirates (Dubai, Ras-Al-Khaimah, Al-Ain)	Cross-sectional	GMS-A3	—	610	Household, random sample of 843 households (>60 yrs)	Cognitive impairment with or without dementia—3.6%

MMSE: modified mini-mental state examination; CPR: crude prevalence rate; MCI: mild cognitive impairment; VaD: vascular dementia; MES: Memory and Executive Screening test; GDS: Geriatric Depression Scale; NINCDS-ADRDA: National Institute of Neurological and Communicative Disorders and Stroke and the Alzheimer's Disease and Related Disorders Association; NINDSAIREN: National Institute of Neurological Disorders and Stroke (NINDS) and the Association Internationale pour la Recherche et l'Enseignement en Neurosciences; SMQ: short-memory questionnaire; BCST: Brookdale Cognitive Screening Test; A-IQCODE 16: Arabic Version of 16-item Informant Questionnaire on Cognitive Decline for the older adults; DRG: Dementia Research Group; MoCA: Montreal Cognitive Assessment; GMS: Geriatric Mental State Interview; CERAD: Consortium to Establish a Registry of Alzheimer's Disease; NEUROEX: physical assessment and brief neurological examination; RUDAS: Rowland Universal Dementia Assessment Scale.

**Table 2:** Hospital-Based Data—Dementia Frequency.

Author	Year	Country	Study design	Criteria used for dementia identification	Sample size	Setting/sampling tech	Study key findings
Ogunniyi et al. [26]	1998	Saudi Arabia	Hospital-based study	Dementia subtypes were made according to NINCDS-ADRDA, NINDSAIREN, and ICD 10	77	King Khalid University Hospital, Riyadh (KKUH) All Saudis with dementia at the university hospital from Jan 1985 to Dec 1996	Hospital frequency—19.3/100,000 Dementia subtypes: AD—51.9% VaD—18.2% Mixed cases—15.6% Dementia with Parkinson's disease—7.8% Treatable dementia—5.2%

Qureshi et al. [27]	2001	Saudi Arabia	Hospital-based study	Not mentioned	540	Hospital, random selection of 540 referrals to psychiatric hospital (1999)	Psychiatrists made more diagnoses of dementia than PHC or GH doctors (not significant) From 138 GH referrals: 9.4% of dementia patients were diagnosed by a psychiatrist 1.4% of dementia patients were diagnosed by the GH psychiatry doctors

Albugami et al. [3]	2018	Saudi Arabia	Hospital-based study	Medical records No standardized diagnostic protocol	418	Dementia patients in Saudi hospital from 1995 to 2010	Dementia subtypes: Mixed dementia—18.37% AD—15.87%. VaD—7.71% Parkinson dementia—3.85%

Hamad et al. [28]	2004	Qatar	Hospital-based study	MMSE, SMQ brain CT, MRI, blood tests	134	Dementia patients Hamad General Hospital, Doha 1997–2003	Dementia subtypes: AD—39 (29%) VaD—30 (22%) Mixed AD and VaD—20 (15%) Dementia with Parkinson's disease—8 (6%)

**Table 3:** Studies on dementia risk factors.

Author	Year	Country	Study design	Outcome measure	Sample size	Setting/sampling tech	Study key findings
Chaaya et al. [29]	2018	Lebanon (Beirut, Shouf, and Aley)	Cross-sectional secondary analysis of Chahine et al. [21]	[1] 10/66 DRG, modified CERAD, animal naming tests modified 10-word list recall, GMS, CSI-D [2] Self-reported on HTN, DM, CVD, smoking, PA [3] 3 consecutive measurements of blood pressures	*N* = 502	Households Multi-stage cluster sampling	Age: OR 75–84 years = 4.00 (95% CI: 1.46, 10.95); OR 85+ years = 7.07 (1.84, 27.03) compared to age group 64-74 years Perceived insufficient income (vs. sufficient income): OR 3.90 (95% CI: 1.58, 9.60) No formal education (vs. formal education): OR 3.39 (95% CI: 1.71, 6.70) Uncontrolled hypertension (vs. no hypertension): OR 6.35 (95% CI: 1.60, 25.10)

Farrag et al. [17]	1998	Egypt (Assiut Governorate)	3-stage cross-sectional	MMSE Clinical diagnosis Lab investigation	2000	Household, systematic random of total elderly $\text{population }\underset{¯}{>}60$ years (*N* = 130,000)	Occupation and residence did not affect the prevalence or severity of dementia Age-specific dementia prevalence tends to double every 5 years after age 75 years and above

Ouanes et al. [24]	2014	Tunis	Cross-sectional	MoCA	77	Manouba nursing home All 116 residents (mean 72.6 years ± 10.3)	Prevalence of dementia did not differ significantly by gender, age, marital status, level of education, profession, current financial situation, or depending on the participation in the activities at the center

El Tallawy et al. [19]	2014	Egypt (Al-Quseir city)	Cross-sectional	MMSE, clinical exam, investigations	8,173 (≥50)	Household All persons (*N* = 33,285)	Prevalence of dementia significantly higher among illiterate than educated participants (3.6% vs. .79%)

Khedr et al. [20]	2015	Egypt	Cross-sectional	MES and MMSE Neuro exam of all positive cases—DSM-IV, Hachinski ischemic score	691	Household, multistage probability random sampling	CPRs were significantly higher in the following: Illiterate than literate participants (10.12 vs. 2.25 cases per 100 population, *p* < 0.001) Urban than rural areas (7.1 versus 3.27%, *p* = 0.03) Industrial than nonindustrial areas (13.23 vs. 1.99; *p* < 0.001)

Al-Khateeb et al. [30]	2014	Jordan	Case control	MMSE, Clock Drawing Test	102	Senior homes and Jordan University Hospital (52 dementia patients and 50 controls) >60 yrs	Risk for dementia: Educational level < 12 years—OR 3.29 (*p* = 0.026) Illiterate—OR 6.29 (*p* = 0.002) compared to education level more than 12 years No significant correlation between serum copper, lipid profile, and cognitive decline in elderly Jordanians Coffee intake has a protective effect against cognitive decline (6.25-fold lower risk)

Alaama et al. [31]	2016	Saudi Arabia (Jeddah)	Cross-sectional	MoCA, RUDAS	241	King Abdulaziz University Hospital volunteers 171 outpatients with DM, matched with 70 controls without DM Age 59.6 ± 9.2 years	Diabetics more likely to have cognitive impairment than nondiabetics (16% vs. 3%; *p* = 0.004). With MoCA, 85% of the cases and 78% of the controls had abnormal results (*p* = 0.194). Among diabetics, there was no statistically significant effect found for glycemic control or DM duration on either test

Haithem et al. [32]	2018	Tunisia	Case control		200 dementia patients and 300 controls	—	Association between dementia risk and all the studied polymorphisms except PON1-Q192R was found to be significant APOE e4 allele—OR 4.32 (*p* = 0.001) ACE I and PON1-L55M T alleles—OR 2.58 and 2.11 (*p* < 0.001 and *p* = 0.015) GTICC haplotype associated with 9-fold dementia risk (*p* < 0.001), whereas AADTT seems to reduce dementia risk by 80% (*p* = 0.003)

Albugami et al. [3]	2018	Saudi Arabia	Retrospective, cohort	No standardized diagnostic protocol	418	Medical records of patients have dementia at tertiary care hospital from 1995 to 2010 Mean age 78.8	Comorbidities: 27.44% of patients have more than 3 risk factor comorbidities High prevalence of mixed dementia could be related to high prevalence of CVD risk factors like hypertension, dyslipidemia, and diabetes mellitus among Saudis Stroke is reported in 15.03%

Shamieh et al. [34]	2018	Lebanon	Cross-sectional		591 individuals	—	Prevalence of APOE genotypes in Lebanon was similar to that seen in Asian populations APOE genotypes not associated with hypercholesterolemia A significant difference between APOE genotypes in AD cases versus controls and versus Lebanese general population was seen E4 allele was approximately threefold higher in Alzheimer's disease study patients when compared with the remaining individuals

MMSE: modified mini-mental state examination; CPR: crude prevalence rate; VaD: vascular dementia; MES: Memory and Executive Screening test; GDS: Geriatric Depression Scale; NINCDS-ADRDA: National Institute of Neurological and Communicative Disorders and Stroke and the Alzheimer's Disease and Related Disorders Association; NINDSAIREN: National Institute of Neurological Disorders and Stroke (NINDS) and the Association Internationale pour la Recherche et l'Enseignement en Neurosciences; SMQ: short-memory questionnaire; BCST: Brookdale Cognitive Screening Test; A-IQCODE 16: Arabic Version of 16-item Informant Questionnaire on Cognitive Decline for the older adults; DRG: Dementia Research Group; MoCA: Montreal Cognitive Assessment; GMS: Geriatric Mental State Interview; CERAD: Consortium to Establish a Registry of Alzheimer's Disease; NEUROEX: physical assessment and brief neurological examination; RUDAS: Rowland Universal Dementia Assessment Scale.

**Table 4:** Quality assessment of the included studies.

Author	Sample size	Study design	Response proportion	Diagnostic assessment	Total
Farrag et al. [17]	1.5	2	3	2	8.5
El Tallawy et al. [18]	2	2	3	4	11
El Tallawy et al. [19]	2	2	3	4	11
Khedr et al. [20]	1	2	3	4	10
Chahine et al. [21]	0.5	2	1	1	4.5
Phung et al. [22]	1	0	1	4	6
Ogunniyi et al. [26]	0.5	0	0	2	2.5
Qureshi et al. [27]	1	N/A	N/A	3	4
Albugami et al. [3]	0.5	2	3	4	9.5
Hamad et al. [28]	0.5	2	0	4	6.5
Ghuloum et al. [23]	1.5	1	2	2	6.5
Ouanes et al. [24]	0.5	0	2	1	3.5
Ghubash et al. [25]	1	2	3	4	10
Chaaya et al. [29]	1	0	1	4	6
Al-Khateeb et al. [30]	0.5	0	0	1	1.5
Alaama et al. [31]	0.5	0	0	1	1.5
Haithem et al. [32]	0.5	0	0	1	1.5
Shamieh et al. [34]	1	0	0	1	2

Sample size: <500, 0.5 point; 500–1499, 1 point; 1500–2999, 1.5 points; ≥3000, 2 points. Study design: two-phase or one-phase study with no sampling of screen negatives, 0 points; two-phase study with sampling of screen negatives but no weighting back, 1 point; one-phase study or two-phase study with appropriate sampling and weighting, 2 points. Response proportion: not mentioned, 0; <60%, 1 point; 60–79%, 2 points; ≥80%, 3 points. Diagnostic assessment: one point each for multidomain cognitive test battery, formal disability assessment, informant interview, and clinical interview.
